# The Pathophysiological Mechanism and Clinical Treatment of Polycystic Ovary Syndrome: A Molecular and Cellular Review of the Literature

**DOI:** 10.3390/ijms25169037

**Published:** 2024-08-20

**Authors:** Kai-Jung Chang, Jie-Hong Chen, Kuo-Hu Chen

**Affiliations:** 1 Department of Obstetrics and Gynecology, Taipei Tzu-Chi Hospital, The Buddhist Tzu-Chi Medical Foundation, New Taipei City 23142, Taiwan; tch36289@tzuchi.com.tw; 2 Department of Medicine, MacKay Medical College, New Taipei City 25245, Taiwan; albertjhc@gmail.com; 3 School of Medicine, Tzu-Chi University, Hualien 97004, Taiwan

**Keywords:** polycystic ovary syndrome, insulin resistance, hyperinsulinemia, hyperandrogenism

## Abstract

Polycystic ovary syndrome (PCOS) is a prevalent metabolic disorder among women of reproductive age, characterized by hyperandrogenism, ovulatory dysfunction, and polycystic ovaries. The pathogenesis of PCOS involves a complex interplay of genetic and environmental factors, including insulin resistance (IR) and resultant hyperinsulinemia. Insulin receptors, primarily in skeletal muscle, liver, and adipose tissue, activate downstream signaling pathways like PI3K-AKT and MAPK-ERK upon binding. These pathways regulate glucose uptake, storage, and lipid metabolism. Genome-wide association studies (GWASs) have identified several candidate genes related to steroidogenesis and insulin signaling. Environmental factors such as endocrine-disrupting chemicals and lifestyle choices also exacerbate PCOS traits. Other than lifestyle modification and surgical intervention, management strategies for PCOS can be achieved by using pharmacological treatments like antiandrogens, metformin, thiazolidinediones, aromatase inhibitor, and ovulation drugs to improve insulin sensitivity and ovulatory function, as well as combined oral contraceptives with or without cyproterone to resume menstrual regularity. Despite the complex pathophysiology and significant economic burden of PCOS, a comprehensive understanding of its molecular and cellular mechanisms is crucial for developing effective public health policies and treatment strategies. Nevertheless, many unknown aspects of PCOS, including detailed mechanisms of actions, along with the safety and effectiveness for the treatment, warrant further investigation.

## 1. Introduction

Polycystic ovary syndrome, often abbreviated as “PCOS”, features a female metabolic disorder that centers around three primary concepts: clinical and biological hyperandrogenism (HA), ovulatory dysfunction (OD)—often manifested as irregular menstruation or amenorrhea—and morphological polycystic ovaries (PCO). Identified as one of the leading yet treatable causes of infertility, it affects roughly 2% to 40% of women at reproductive age, and more generally, between 6% to 13% of women overall, according to most studies. The estimated prevalence rate may differ depending on the diagnostic criteria, ethnicity, study method, and others [1,2,3,4]. For example, a paper published in 2018 stated that based upon the National Institutes of Health (NIH)’s diagnostic criteria, the United States shared a similar prevalence rate with some European countries. On the other hand, some geographical differences have been pointed out in previous studies. Furthermore, there was no conclusive answer to date whether a clear relation between regional differences, ethnicity and the prevalence of PCOS could be clarified [5].

**Figure 1:**
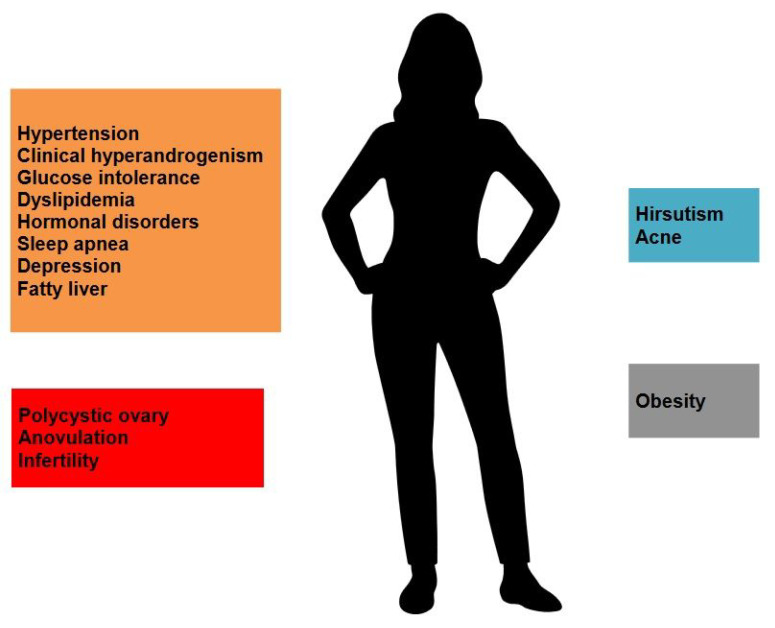
The clinical presentations and comorbidities of PCOS.

In addition, some also believe that ethnic differences have an impact on the phenotype of PCOS. For instance, several studies have suggested that Hispanic women tend to have a more severe phenotype than non-Hispanic black women [6]. There is also existing data suggesting that East Asian women with PCOS are more prone to metabolic syndrome despite relatively lower risk for obesity, while hyperandrogenic manifestations are more likely to be observed among the Middle Eastern and Mediterranean counterparts [7]. In short, the clinical phenotype of PCOS has great diversity among regions, races, and countries, and hence its manifestations may vary greatly within the spectrum of HA, endocrinal and metabolic disorders (Figure 1).

**Figure 2:**
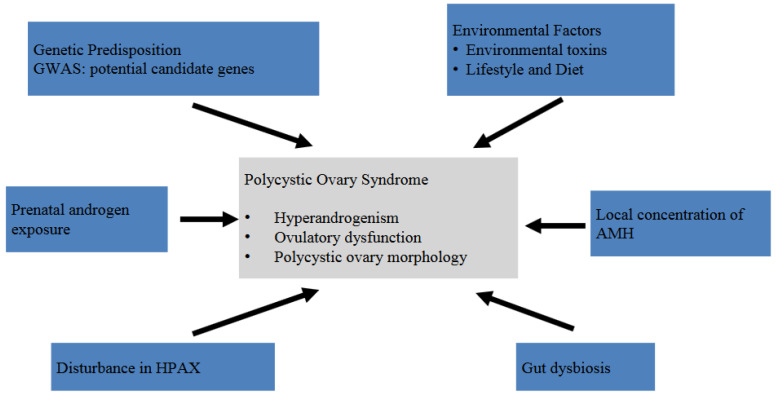
The etiology, pathophysiology, and hallmarks of PCOS.

Considered as a multifactorial disorder, PCOS is affected by risk factors consisting of a great variety of genetic predisposition, particular physical conditions such as gut dysbiosis or diabetes mellitus, lifestyle, family history, and environmental factors [8,9]. Figure 2 is a brief summary showing the etiology, pathophysiology, and hallmarks of PCOS. PCOS is usually diagnosed during adolescence or adulthood. There is currently a lack of strong evidence and valid criteria regarding its diagnosis after menopause [1,10]. Female patients may make their encounters to medical facilities due to various clinical manifestations or consequences related to PCOS without having a previously confirmed diagnosis. Common symptoms and signs include irregular menstruation, chronic pelvic pain, masculine features (such as acne, alopecia, hirsutism, etc.) and infertility [2]. However, it may be under- or over-diagnosed if an impression is made by an almost “instinctive” clinical judgement since there are many conditions that mimic PCOS. Therefore, it is suggested to follow a widely accepted guideline or consensus, even though the diagnostic criteria still differ in between. Currently, most agree that the Rotterdam criteria, established in 2003, are probably the most commonly applied diagnostic standard [1,11].

PCOS is a complex disorder of which various theories regarding its etiology and pathophysiology have been proposed. Given its shared traits with some metabolic disorders such as obesity and diabetes mellitus, a similar disease mechanism seems to be plausible. In fact, insulin resistance (IR) is one of the key elements to the genotypes and phenotypes of PCOS [12]. IR results in hyperinsulinemia and HA, leading to a series of cellular reactions that reflect on the physical traits of PCOS. Genome-wide association studies (GWASs) have identified many candidate genes, although specific mutations still remain elusive due to the heterogeneity of PCOS [13]. On the other hand, environmental factors such as endocrine-disrupting chemicals (EDCs), and certain lifestyle and dietary habits may induce or exacerbate PCOS-like traits, too. The influence of EDC exposure may begin as early as the beginning of gestation [14,15]. The interaction between genetic predisposing factors and environmental factors induces the hallmarks of PCOS (Figure 2). For instance, when a gene variation causes dysregulation in androgen biosynthesis or glucose metabolism, HA and IR may result [16,17].

Popular management strategies include lifestyle modification, medication, and other types of non-pharmacological therapies such as alternative medicine and hair removal for patients with hirsutism. Managements do not cure or reverse PCOS but may ameliorate the annoying symptoms and complications related to PCOS. Lifestyle and diet modification serve as the primary approach to managing PCOS. Pharmacological interventions such as including antiandrogens and metformin may deal with the hyperandrogenic features and metabolic disorders associated with PCOS. These treatment strategies aim to improve insulin sensitivity and restore normal ovulation induction [1].

According to an article published by Carrie Riestenberg et al. in 2021, it is estimated that PCOS brought about an economic burden of USD 8 billion in 2020, with approximately USD 3.7 billion contributing to an initial diagnosis or management of associated reproductive endocrine disorders [18]. Given the considerable prevalence and economic burden of PCOS, a comprehensive understanding of the disorder is crucial for introducing public health policies, improving treatment strategies, and ultimately improving life quality for individuals affected by PCOS. Our study aims to provide a thorough review on the etiology, pathophysiology, diagnosis, and management regarding PCOS, with an emphasis on the molecular and cellular aspect of its pathophysiology and management.

## 2. Research Method and Literature Review

**Figure 3:**
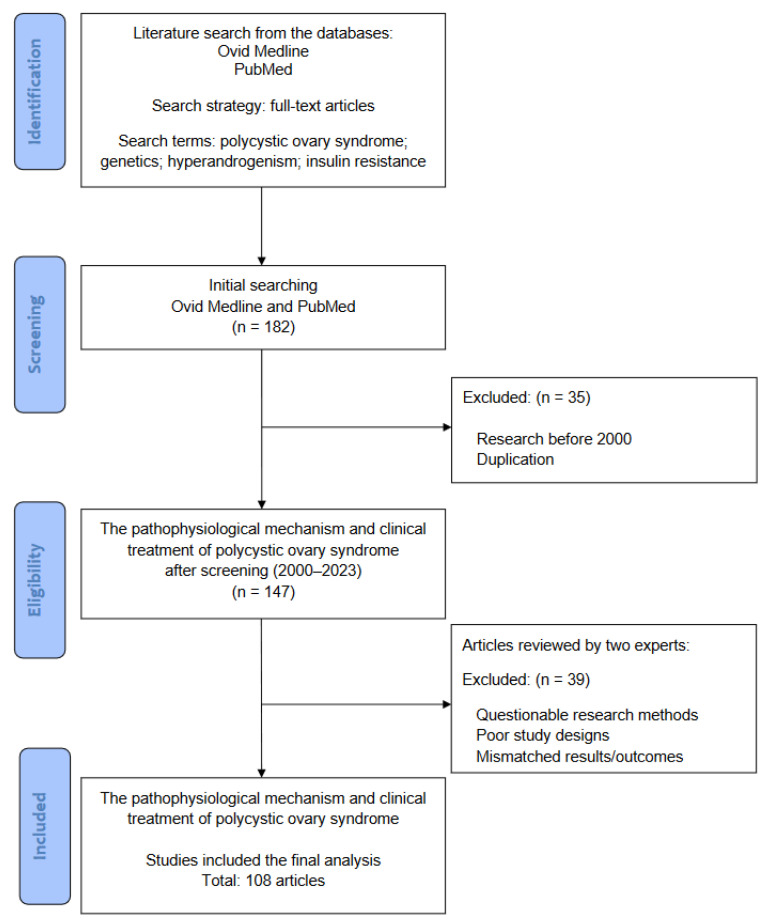
The flowchart of database searching, screening, selection, and inclusion of eligible articles from the literature.

In this review, the literature was searched to solicit basic and clinical studies that investigated the underlying molecular and cellular mechanisms of PCOS, including the genetic, hormonal, and pathophysiological changes and environmental effects, as well as its treatment. The literature review was performed between September 2023 and March 2024. Figure 3 illustrates the flowchart of database searching, screening, and inclusion of the references that we selected from the literature. In the first stage, all of the studies were collected from the databases Ovid Medline and PubMed using the search terms “polycystic ovary syndrome”, “genetics”, “hyperandrogenism”, and “insulin resistance” for the research topic. For screening and selection in the next stage, only full-text articles were considered to be included for a further analysis. In the second stage, research published before 2000 was excluded to ensure the novelty of the review. At the same time, duplicated articles were also excluded. From a total of 182 articles identified in the screening process, 147 articles (2000–2023) met the criteria for further inclusion.

In the next stage, two experts in the field independently inspected the contents of articles such as research materials, study designs, results, and/or outcomes to identify eligible studies for further inclusion. The collected articles with questionable research methods, poor study designs, or mismatched results/outcomes were excluded at this stage. Discrepancies between experts were discussed by mutual communication to reach a consensus. All eligible articles were included in this review using the abovementioned search terms and strategies (database searching, screening, selection, and inclusion of articles). Finally, a total of 108 articles were retrieved for the current review.

## 3. Diagnosis of PCOS

Hyperandrogenism, ovulatory dysfunction, and morphological polycystic ovaries are the three primary features of PCOS. To date, there are three acknowledged sets of diagnostic criteria: the NIH criteria, the Rotterdam criteria, and the Androgen and Excess and PCOS Society (AE-PCOS) criteria. These diagnostic guidelines center on the three core clinical manifestations of PCOS, but with differences in emphasis and severity [1,19]. Table 1 is a comparison between different diagnostic criteria for PCOS.

### 3.1. The NIH Criteria

Stein and Leventhal were the first ones to describe seven female cases with the classic characteristics of the syndrome later known as PCOS in 1935. It was not until 1990, however, that the National Institute of Child Health and Human Development made an attempt to provide a more precise definition for PCOS [11]. The NIH became the first organization to develop standard and formal diagnostic criteria in 1990, and the prevalence rate of PCOS is around 5–8% based on this guideline. The NIH suggests two classic conditions as a key to PCOS: irregular or absent ovulation/menstruation (OD) and biochemical or clinical evidence of hyperandrogenism. Women need to be identified with both conditions to be diagnosed with PCOS, while pelvic imaging of polycystic ovaries is not an essential NIH criterion since not all women with other PCOS features are found with them [20]. There are two possible phenotypes of PCOS based on the NIH criteria: HA + OD and HA + OD + PCO.

### 3.2. The Rotterdam Criteria

In 2003, experts gathered in Rotterdam, the Netherlands, where they reached a “Rotterdam consensus”—later known as the “Rotterdam criteria”—regarding the diagnosis of PCOS, which has remained the most well-known and recommended guideline. In fact, the Rotterdam criteria have been widely applied over the recent twenty years [11,21]. It suggests that to define PCOS, two out of the three classic phenotypes—OD, HA, and PCO, defined by at least 12 follicles at the size of 2–9 mm in diameter or an ovarian volume > 10 cm^3^ in one or both ovaries—are required [11,20]. Depending on different combinations of the three diagnostic criteria, PCOS could be classified into four subtypes with different phenotypes. A full-blown or frank phenotype, defined as phenotype A, infers PCOS with the features of HA + OD + PCO. Phenotype B describes non-PCO PCOS with HA + OD. Ovulatory PCOS, which is referred to as phenotype C, includes traits of HA + PCO. Finally, phenotype D, also known as non-hyperandrogenic PCOS, indicates patients who present with OD + PCO. Phenotype A consists of more than 60% of PCOS cases. It is associated with relatively unfavorable metabolic and cardiovascular outcomes, and also a higher clomiphene resistance. On the other hand, phenotype D is the least severe type. Although not all demographic, hormonal, or metabolic parameters present with a significant difference among different phenotypes, understanding the diverse nature of PCOS may provide a better picture of the disease severity and fertility outcomes of different patients [22]. In-depth studies have shown that, in contrast to some previous viewpoints, such as the consensus of the NIH criteria, the PCO morphology should be considered as a significant marker of PCOS [23]. Overall, the prevalence rate of PCOS according to the Rotterdam consensus could be as high as 15% to 18% [11,24].

### 3.3. The AE-PCOS Criteria

The AE-PCOS criteria argue that HA is crucial to the diagnosis of PCOS since it is linked to a more serious metabolic outcome. In addition, either PCO or OD is required for a definite diagnosis. Hence, there are three possible combinations to meet the criteria: HA + PCO, HA + OD, or HA + OD + PCO [1]. For making the diagnosis, other disorders or confusing conditions that may mimic PCOS such as 21-hydroxylase deficient non-classic adrenal hyperplasia, androgen-secreting neoplasms, androgenic/anabolic drug use or abuse, Cushing’s syndrome, Hyperandrogenic-IR-Acanthosis Nigricans syndrome, thyroid dysfunction, and hyperprolactinemia should also be excluded [25]. The prevalence rate of PCOS using the AE-PCOS criteria is similar to or slightly lower than that of the Rotterdam criteria [26,27].

To date, there have been numerous studies to investigate different criteria and phenotypes of PCOS. This furthermore proves the complexity of this disorder and explains why its disease mechanism and pathophysiology continue to provoke interests in experts, which will be discussed in detail in the following section.

## 4. Pathogenesis and Pathophysiology

The pathogenesis of PCOS could be viewed as a complicated and multifactorial interplay between genetic and environmental factors. In our review, we aim to investigate previously proposed theories in the hope of better understanding its disease mechanism and pathophysiology from a molecular and cellular perspective.

An article published by the *International Journal of Molecular Sciences* in 2020 proposed a conceptual diagram, suggesting that an interaction between the environment and genetics leads to HA, IR, hyperinsulinemia, and metabolic abnormalities. As described in the following paragraphs, the interaction may take place as early as the gestational period and result in lifelong influences in the offspring [28].

### 4.1. Genetic Predisposition

The high familial aggregation of PCOS strongly implicates a genetic impact on this particular metabolic condition. In essence, many studies have proven this viewpoint. Contrary to a common misbelief that a genetic linkage or specific gene mutation could be found in a family, however, this is not the case most of the time, owing to the complexity and heterogeneity of PCOS [13]. Nevertheless, GWASs may provide substantial help in identifying some candidate genes in a wider population. Any genes that directly or indirectly affect the ovaries and metabolic system could be related to PCOS; hence, a wide variety of genes have been hypothesized to be associated with its pathogenesis [13,29,30].

#### 4.1.1. The Genetics of PCOS

The likelihood of the inheritance of PCOS was first proposed in 1968. Since then, there has been rising evidence regarding the familial linkage and genetic basis of PCOS, with some suggesting it as an autosomal dominant or X-linked disorder. Some of its representative traits, hirsutism and OD, for instance, have also been suggested to be monogenic. However, due to its extreme complexity and polygenic causes, linkage analysis usually results in negative findings, even within a family [30]. So far, the candidate genes suggested by GWASs could only account for less than 10% of its inheritance [31].

The limitations of GWASs in the research for PCOS have been further illustrated by a study published by Danielle Hiam, Alba Moreno-Asso, Helena J. Teede et al. First, some potentially real yet weak gene associations may be masked by statistical adjustments. Second, a GWAS is not sensitive enough to identify rare genetic variants including SNPs [30]. Therefore, aside from GWAS, which has failed to identify any definite single candidate gene to date, other approaches such as intrauterine programming have been introduced [13]. Phenome-wide association study (PheWAS) is another approach, which involves examining different genetic variants that may be associated with different phenotypes of PCOS [30]. Whole-exome sequencing (WES), which explores the whole exome and can process massive parallel sequencing, may also provide information about rare variants [31]. It is also worth noting that a geological association should warrant more importance than merely an ethnic or racial relation, since the former is a relatively more accurate predictor of ancestry [30].

That said, nevertheless, GWAS remains to be one of the primary research measures for the genetic predisposition of PCOS. Candidate gene study is another common method, which refers to an approach to identify possible SNPs and validate the study results of GWASs [32]. The candidate genes involved in PCOS will be discussed in more detail in the next section.

Besides the primary or genetic variants, epigenetics is also a key factor to the pathogenesis of PCOS. Although not directly related to DNA changes, the epigenetic changes are responsible for the upstream regulation of genetic behaviors. The importance of epigenetics—CPG island methylation and histone modification in particular—in PCOS-like manifestations has been demonstrated in animal models. Both prenatal and peripubertal dietary influences play a role in epigenetics, once again asserting the interplay between environmental and genetic factors in PCOS [32].

#### 4.1.2. The Candidate Genes for PCOS

Despite the fact that no single gene is identified to have a direct causative relation with PCOS, scholars have proposed some candidate genes that may contribute to its manifestations, such as genes involved in the metabolism of androgen and function of insulin.

The investigation possibly dates back to the late 20th century. In 1999, Margrit Urbanek et al. published a paper regarding 37 possible candidate genes. The study suggested CYP11A and follistatin—which is associated with the arrest of follicular development and reduction in FSH—to have a possible yet probably not solid linkage [33]. A paper published in 2001 provided a review regarding possible candidate genes related to LH production, LH receptors, androgen biosynthesis, insulin secretion, insulin receptors, and folliculogenesis. The study reached a conclusion that CYP11A and follistatin may play a role in the pathogenesis of PCOS but without a definite causative relationship. No other candidate genes have proven such strong evidence [34].

In more recent studies, GWAS became a much more common approach to identify potential genetic risk factors. New evidence has suggested that variance in the *DENND1A* (Differentially Expressed in Normal and Neoplastic Development isoform A1) gene, which encodes the *DENND1A* protein located in theca cells, may be related to PCOS owing to its involvement in steroidogenesis. Other plausible candidate genes include LHCGR (associated with LH levels and hyperandrogenism), FSHR (associated with ovarian response to FSH), INRS (associated with insulin resistance), etc. [31]. The CYP family remains an important target of study, but a firm direct association could not be entirely established. On the other hand, genes involved in LH and FSH function may have an indirect role in the pathogenesis of PCOS. The *AMH* gene may also be a strong predictor. Other possible candidate genes include *FTO*, *PCOS 1*, *SRD5A1*, *SRD5A2*, etc. [13]. However, none of these candidate genes could explain more than 10% of the heritance linkage of PCOS. Therefore, it is also very common for an identified candidate gene to fail to replicate its genetic influence in another study.

### 4.2. Hyperandrogenism

#### 4.2.1. Steroidogenesis

Steroid biosynthesis and metabolism entail complex pathways that take place in many organs and bodily tissues. In our review, we focus on the cellular and molecular reactions of ovarian and adrenal steroidogenesis in women at reproductive age.

**Figure 4:**
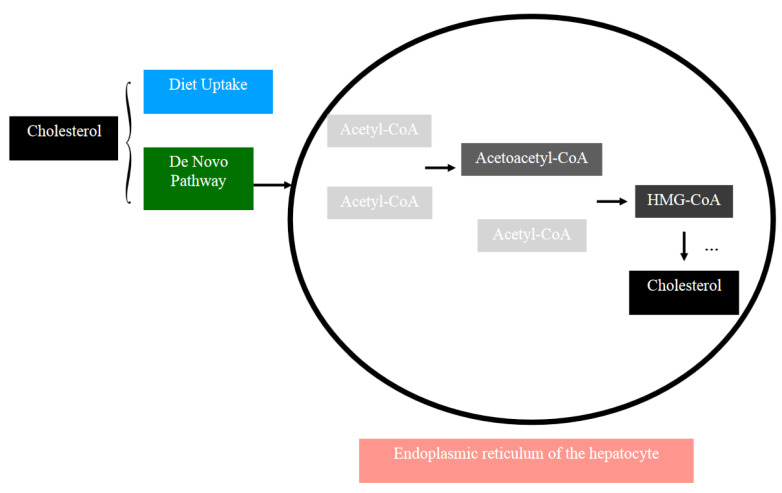
A summarized process of the biosynthesis of cholesterol. Cholesterol is either absorbed through diet uptake or more often produced by the de novo pathway, which takes place at the endoplasmic reticulum of the hepatocyte (the arrows depicted a cascade of the step-by-step biosynthesis). The initial reactants are two acetyl-CoA molecules, which merge and convert into acetoacetyl-CoA. Acetoacetyl-CoA then merges with another acetyl-CoA molecule to form HMG-CoA. HMG-CoA goes through a series of complex chemical reactions (displayed as “…”) and is eventually converted to cholesterol.

Steroidogenesis begins with cholesterol as the primary reactant. Cholesterol is constructed with a three-domain structure, the hydrophilic, hydrophobic, and rigid domain, respectively. This characteristic grants it the ability to be engaged in various cellular activities, making it one of the most important regulatory molecules of the human body, and also the precursor of steroid hormones [35]. Cholesterol could be synthesized in the de novo pathway or be absorbed by the body through diet uptake, and the majority of its synthesis through the de novo pathway is contributed by the liver. The reaction occurs in the endoplasmic reticulum (ER), in which two acetyl-coenzyme A (acetyl-CoA) molecules merge into acetoacetyl-CoA and combines with another acetyl-CoA to become 3-hydroxy-3-methylglutaryl CoA (HMG-CoA). HMG-CoA is reduced to mevalonate and is eventually converted into cholesterol after almost 30 reaction steps that include phosphorylation, decarboxylation, polymerization, and others [36]. Figure 4 illustrates the biosynthesis of cholesterol.

Following its synthesis, cholesterol is then transported to the outer mitochondrial membrane (OMM) and later moved into the inner mitochondrial membrane (IMM) by the steroidogenic acute regulatory (StAR) proteins that are responsible for transcellular lipid transfer in mammals. The key enzymes to steroidogenesis are cytochrome P450s (CYPs) and hydroxysteroid dehydrogenases (HSDs). Most of these enzymes trigger unidirectional and irreversible reactions. CYPs catalyze oxidation by receiving electrons from nicotinamide adenine dinucleotide phosphate (NADPH) [37].

**Figure 5:**
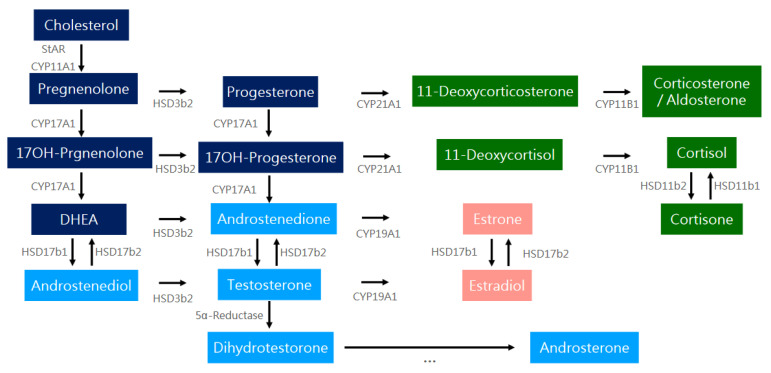
The process of human steroidogenesis. Through a series of different chemical reactions, cholesterol can be converted into cortiocosterone/aldosterone, cortisol and sex hormones, respectively. The process is delicately regulated by various catalysts and signaling pathways. The arrows indicate the direction of the biosynthesis, catalyzed by different enzymes. Androgens such as androstenedione and testosterone can be converted into female sex hormones such as estrone and estradiol by CYP19A1. Dihydrotestosterone can be converted into androsterone through a series of different reactions with multiple catalysts involved, shown with “…” below the lowest arrow.

Initially, CYP11A1 catalyzes the conversion of cholesterol into pregnenolone. Pregnenolone is converted into progesterone by HSD3b2. Progesterone can undergo a further conversion into 11-deoxycorticosterone by CYP21A1, and then into corticosterone by CYP11B1. Another corticoid synthesis pathway leads from 17-OH progesterone, derived from progesterone by CYP17A1, to 11-deoxycortisol via CYP21A1, and then to cortisol by CYP11B1. Cortisol and cortisone can be transformed into each other by HSDs. In addition, 17-OH progesterone is also a precursor of androgens. With the aid of CYP17A1, it can be converted into androstenedione, and later into testosterone and dihydrosterone through a series of reactions, catalyzed by HSD17b and 5α-reductase, respectively. Androgens may be converted into estrogens (estrone and estradiol) by CYP19A1, also known as the aromatase. Estrogen and estradiol can convert each other by the HSD family. Pregnenolone, on the other hand, can be converted into 17-OH pregnenolone and further into DHEA by CYP17A1. DHEA can be converted into androstenediol by HSD17b1. 17-OH pregnenolone, DHEA, and androstenediol can all be converted into 17-OH progesterone, androstenedione, and testosterone, respectively, by HSD3b2, contributing to the androgen and estrogen synthesis pathways. The hemostasis of this complex network is crucial for maintaining the levels of steroid as well as sex hormones and responding to physiological demands [37,38]. The process of human steroidogenesis is showed in Figure 5.

**Figure 6:**
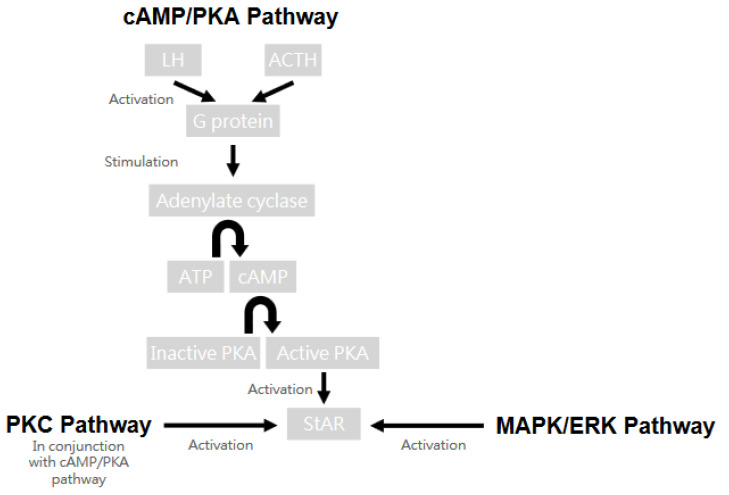
The cell signaling pathway of steroidogenesis.

There are numerous signaling pathways that regulate the complicated process of steroidogenesis intricately, and many of them center around the expression of StAR. One of the most important ones is the cAMP/PKA pathway. This pathway describes the scenario where luteinizing hormone (LH) and adrenocorticotropic hormone (ACTH) activate G proteins, leading to the stimulation of adenylate cyclase and the production of cyclic adenosine monophosphate (cAMP). Like a cascade, cAMP subsequently activates protein kinase A (PKA), which further phosphorylates and activates important proteins, including StAR. The protein kinase C (PKC) pathway may also activate StAR in conjunction with the cAMP/PKA pathway but does not have the ability alone. Arachidonic acid (AA) and its metabolites also play an important part in steroid biosynthesis by modulating the StAR gene expression. Metabolites generated through the lipoxygenase and epoxygenase pathways typically upregulate steroidogenesis, while metabolites that come from the cyclooxygenase 2 (COX2) pathway have a tendency to downregulate the process. In addition, growth hormones such as epidermal growth factor (EGF) and insulin-like growth factor I (IGF-I) are also crucial to steroid synthesis. They boost steroidogenesis and StAR expression through several pathways like the MAPK/ERK pathway. Last but not least, electrolytes, namely, chloride and calcium ions, are also involved in the signaling pathways. Chloride ions affect steroidogenesis at physiological LH levels by affecting relevant enzymes, while calcium ions may function as second messengers, influencing signaling pathways and key enzymes directly or indirectly [30,37,39,40]. The cell signaling pathway of steroidogenesis is depicted in Figure 6.

#### 4.2.2. Genes Involved in Hyperandrogenism

##### CYP11A

The CYP11A locus plays an important role in steroid synthesis, of which a variation is significantly affiliated to HA. It encodes cytochrome P450 side-chain, which cleavages cholesterol and converts it into pregnenolone, which is subsequently turned into androgen and estrogen after a series of cellular reactions. In other words, CYP11A triggers the first step of steroidogenesis. Hence, an overexpressed gene may result in androgen excess and PCOS characteristics. For example, a CYP11A 5′ UTR pentanucleotide repeat polymorphism has been found to be associated with PCOS patients with hirsute traits [16,41].

##### CYP17

After pregnenolone is produced, it is then hydroxylated by cytochrome P450 17α-hydroxylase to become 17α-hydroxypregnenolone (17α-OHpregnenolone). In another pathway, where pregnenolone is converted by type II 3β-hydroxysteroid-Δ5-steroid dehydrogenase (HSD3b2) to progesterone, cytochrome P450 17α-hydroxylase is involved in the following process of turning progesterone into 17-OH progesterone. CYP17 is the gene that encodes 17α-hydroxylase, and, therefore, a dysregulation or overexpression of this gene may facilitate HA. Previous papers have supported the idea that CYP17 polymorphism is linked to an increased risk of PCOS and phenotypes related to androgen excess such as hirsutism and alopecia [41,42,43].

##### CYP19

CYP19 encodes aromatase, which is the catalyst that is involved in the process of androgen conversion into estrogen. Therefore, it appears to be plausible that a gene variation in CYP19 has a relation with aromatase dysfunction and androgen excess in women [44,45,46]. In a study carried out by Nectaria Xita et al., the (TTTA)n polymorphism of CYP19 was investigated. It was found that fewer repeats and shorter alleles of CYP19(TTTA) may contribute to PCOS phenotypes even though it may not be a major contributor [47]. Similarly, the rs2414096 single-nucleotide polymorphism (SNP) of the CYP19 gene was found to be associated with higher levels of androgens—total testosterone and androstenedione—in women with PCOS in another study [45]. In conclusion, a lower expression or variation in the CYP19 gene may be responsible for androgen (testosterone and androstenedione) excess and corresponding estrogen (estradiol and estrone) deficiency.

##### CYP21

CYP21 is the gene that encodes steroid 21-hydroxylase (21-OHase), which catalyzes the conversion of progesterone into deoxycorticosterone and the conversion of 17-OH progesterone into 11-deoxycortisol. Congenital adrenal hyperplasia is a famous example of 21-hydroxylase deficiency [48]. On the other hand, a CYP21 mutation or variant at the other end of the spectrum could be engaged in PCOS features due to effects of androgen excess. The G972R variant of the IRS1 gene is one example of a CYP21 mutation, though its role in PCOS development may be limited [49,50].

##### Brief Summary of CYP Genes

CYP genes play a significant part in steroidogenesis. Particularly, the polymorphisms of the CYP family could bear effects of androgen excess and result in hyperandrogenic phenotypes in PCOS [16]. It is worth noting that, aside from CYP genes, any genes directly or indirectly related to androgen transportation and utilization, androgenesis, or steroidogenesis may also have a role. The sex hormone-binding globulin (SHBG) gene located on chromosome 17p and the androgen receptor (AR) gene located on chromosome X are some examples. When a mutation or variation emerges in the AR gene, elevated androgen levels may be found as a result of decreased binding. As for SHBG, it is produced by liver cells and binds to androgens such as testosterone to function as a hormone carrier. If a gene variation such as SNP causes lower levels of SHBG, serum androgen rises. The relation between a low SHBG level and PCOS has been established in previous studies as well [13].

### 4.3. Hyperinsulinemia and Insulin Resistance

#### 4.3.1. Insulin Signaling Pathways

Insulin plays a major role in the hemostasis of human metabolism. It is composed of two polypeptide chains—chain A and chain B, 21-residue and 30-residue, respectively—that are linked by two disulfide bonds. It is produced on the islet of Langerhans located within pancreatic β-cells. The insulin gene is initially transcribed and translated into message signals to produce its precursor protein “preproinsulin”, which can be cleaved into “proinsulin”. Proinsulin has approximately one-tenth the biologic effect of insulin. It is subsequently transported into the Golgi apparatus and converted into equimolar amounts of insulin and C peptide [17,51,52,53]. Compared to insulin, C peptide is biologically inactive and has a longer half-life, allowing for a relatively more sufficient time window for testing. Clinical applications include urinary C peptide (UCP) testing, serum C peptide testing, random non-fasting sampling, glucagon-stimulated C peptide test (GST), etc., to evaluate the function of β-cells [54].

The target receptors for insulin are tyrosine protein kinases that are located primarily in the cell membranes of skeletal muscle, liver, and white adipocytes. Receptor tyrosine kinases (RTKs) are single-chained transmembrane polypeptides with an intracellular tyrosine kinase domain that is usually activated via ligand binding. Likewise, through the binding between insulin and the two subunits of its receptor, strongly affinitive crosslinking is created to trigger downstream signaling pathways. No precise model of the insulin-RTK complex has been established to date, and its signal transduction has yet to be fully unveiled. The more widely accepted theory is that once the insulin receptor is activated by phosphorylation, binding sites for signaling proteins emerge and create bonds with insulin receptor substrates (IRSs), initiating intracellular signaling cascades in term [55,56].

**Figure 7:**
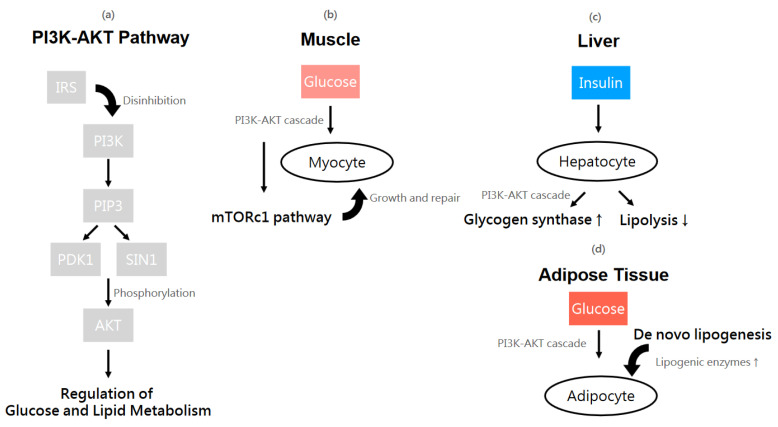
The PI3K-AKT cell signaling pathway of insulin and glucose utilization, which is one of the most significant intracellular signaling cascades involving phosphorylation of mediators (**a**). The muscle (**b**), liver (**c**) and adipose tissue (**d**) are the three main target organs of insulin action. Insulin enhances protein synthesis via the mTORC1 pathway, which facilitates muscle growth and repair (**b**). Once the PI3K-AKT pathway is triggered, it leads to the activation of glycogen synthase and in turn the formation of glycogen in hepatocytes. Concomitantly, insulin suppresses lipolysis and encourages fatty acid synthesis (**c**). Insulin can also enhance lipid storage, known as de novo lipogenesis—through mechanisms such as upregulation of the activity of lipogenic enzymes like fatty acid synthase and acetyl-CoA carboxylase and the inhibition of the breakdown of triglycerides. The arrows in the flow chart are used to describe the directions of the chemical reactions in the signaling pathway. The ↑ and ↓ arrow symbols, displayed next to glycogen synthase, lipogenic enzymes and lipolysis refer to an increase in glycogen synthase and lipogenic enzymes, as well as decrease in lipolysis, respectively.

The main intracellular signaling cascades are the PI3K (phosphatidylinositol 3-kinase)-AKT (v-akt murine thymoma viral oncogene homolog) pathway and the MAPK (mitogen-activated protein kinase)-ERK (extracellular signal-regulated kinase) pathway. PI3K consists of a catalytic subunit that is usually inhibited by a regulatory subunit and only disinhibited when an IRS-1 or IRS-2 binds to the regulatory subunit, subsequently resulting in the production of phosphatidylinositol-3,4,5-triphosphate (PIP3). PIP3 indirectly activates AKT, which is engaged in most of the crucial metabolic effects of insulin. The MAPK-ERK pathway, on the other hand, results from the GRB2 (growth factor receptor bound protein 2)-SOS (son of sevenless Ras/Rac guanine nucleotide exchange factor) complex that binds to IRS or SHC (SH3-containing protein) [56,57]. The PI3K-AKT cell signaling pathways of insulin is presented in Figure 7a.

#### 4.3.2. Insulin Function and Glucose Utilization in Different Sites of Human Body

Insulin functions in various bodily tissues. The main targets are the skeletal muscle, liver, and adipose tissues. Skeletal muscle is a glucose-uptaking tissue that requires energy to maintain its function. The importance of insulin receptors in myocytes has been demonstrated in a rodent model. In muscle-specific insulin receptor knockout mice, for instance, cases with obesity, dyslipidemia, impaired glucose uptake, and glycogen synthesis were observed, although there is no consensus whether or not elevated blood sugar or hyperinsulinemia would result. In contrast, similar experiments in insulin receptor knockout rodent hepatic tissues exhibit a more prominent elevation in insulin secretion and elevated glucose levels. The adipose tissue is also highly sensitive to insulin, in which insulin primarily functions via the PI3K-AKT pathway [55,57].

To delve into further details, the skeletal muscle is the largest human organ that plays a significant role in glucose metabolism, insulin-regulated glucose uptake in particular. The process is highly dependent on the translocation of the insulin-regulated glucose transporter “GLUT4” to the cell membrane, which is a result of the PI3K-AKT cascade. Once the translocation of GLUT4 takes place, glucose enters the myocyte via the channel of translocated GLUT4 on the cell membrane. In addition, insulin activates glycogen synthase and inhibits glycogen phosphorylase, consequently promoting glucose storage as glycogen. Moreover, insulin also enhances protein synthesis via the mTORC1 pathway, which facilitates muscle growth and repair [58,59]. The mTORC1 pathway of glucose utilization in myocytes is presented in Figure 7b.

The liver is responsible for gluconeogenesis (glucose production) and glycogenesis (glucose storage). The role of insulin in hepatocytes is to suppress the former and promote the latter. Once the PI3K-AKT pathway is triggered, the activated AKT inhibits enzymes involved in gluconeogenesis—such as phosphoenolpyruvate carboxykinase (PEPCK) and glucose-6-phosphatase (G6Pase)—through phosphorylation. As glycogen synthase kinase 3 (GSK-3) is also phosphorylated and inhibited by AKT, the inhibitory effect of GSK-3 on glycogen synthase is relieved. This leads to the activation of glycogen synthase and in turn the formation of glycogen. Concomitantly, insulin suppresses lipolysis and encourages fatty acid synthesis, thereby lowering the risk of excessive triglyceride accumulation and hepatic steatosis. In clinical practice, hepatic IR is found in cases with type 2 diabetes mellitus (T2D) and nonalcoholic fatty liver disease (NAFLD) [60,61,62]. The pathway of glucose utilization in hepatocytes is presented in Figure 7c.

As for the adipose tissue, which is mainly composed of adipocytes, insulin again modulates its function through the translocation of GLUT4 to the cell membrane, promoting glucose uptake in a similar manner as in the skeletal muscle. In addition, insulin may enhance lipid storage—known as de novo lipogenesis—through mechanisms such as upregulation of the activity of lipogenic enzymes like fatty acid synthase and acetyl-CoA carboxylase and the inhibition of the breakdown of triglycerides. Insulin may also boost adipokine secretion, although there is no consensus yet [63,64]. The pathway of glucose utilization in adipocytes is presented in Figure 7d. When factors such as obesity, inflammatory reactions, and lipid accumulation cause overloading and disruption in insulin signaling, IR may occur. Targeting crucial components of the IRS-induced PI3K-AKT pathway in skeletal muscle may be one of the keys to developing therapies for IR and T2D.

#### 4.3.3. Genes Involved in Hyperinsulinemia and Insulin Resistance (IR)

Despite strong evidence of IR related to personal lifestyle, some people may bear a genetic predisposition in the first place. In fact, GWAS has identified approximately 60 to 110 loci that may be related to insulin sensitivity, insulin secretion, IR, and T2D according to different studies [65,66,67]. In reviewing various studies, the GENESIS consortium is by far the only one conducted in the scenario of hyperinsulinemia, which suggests NAT2 and the rs1208 loci to be possible targets. On the other hand, the Meta-Analyses of Glucose and Insulin-related traits Consortium (MAGIC) identified loci close to GCKR and IGF1 to be related to IR. Practically, there are dozens of other genetic loci that are related to IR, including many SNPs. Candidate genes derived from different GWASs often vary owing to different definitions of metabolic syndrome [65]. Among those with IR, there is a particular group of people with “severe IR” with an unusually severe IR unproportional to the extent of obesity. Severe IR is often associated with a single gene mutation; however, its pathogenesis has not been clearly identified [68].

### 4.4. Hyperandrogenism (HA), Insulin Resistance (IR), and PCOS

Given the significant familial aggregation of PCOS, many candidate genes of PCOS have been investigated by GWAS, including genes related to gonadotropin release and ovarian function (e.g., FSHB, LHCGR, AMH, DENND1A), genes related to metabolism (e.g., THADA, INSR), etc. Interestingly, these genes do not overlap much with the candidate genes related to HA or IR, and only contribute to a very trivial percentage of the heritability of PCOS. In addition to the genetic predisposition of PCOS, environmental factors play an even more important role in its pathogenesis. Possible factors include intrauterine androgen-rich environment during the prenatal period, proinflammatory follicular environment caused by local concentration of Anti-Müllerian Hormone (AMH) and systemic metabolic abnormalities, exogenous toxins, certain lifestyle preferences, and others. The strong impact of prenatal androgen exposure could be manifested by the fact that these offspring are prone to anogenital anomalies. PCOS-like features and also dysbiosis have been characterized in the rodent family. As mentioned earlier in this article, gut dysbiosis may pose a threat to PCOS. It is likely that gut dysbiosis itself may be a cause for PCOS as well. Hence, interventional therapy such as dihydrotestosterone (DHT) in pregnant women may be a potential preventative measure for PCOS in prenatally androgenized offspring [69].

External factors that cause disturbance in the hypothalamic–pituitary–ovarian axis (HPAX) may also bring negative impacts on the ovarian function. For instance, abnormality in gonadotropin-releasing hormone (GnRH) pulsatility may lead to an excess of LH, which subsequently increases androgen secretion and oocyte development. On top of that, the abnormal levels of downstream hormones disrupt the negative feedback systems and further result in further disturbance of the HPAX [70].

Not only are LH and androgen elevated in women with PCOS, but their sensitivity to these hormones also increases simultaneously. Some believe that hyperinsulinemia, which is secondary to IR, contributes to LH hypersensitivity, too. Previous studies have also suggested that intrinsic theca cell defects might be an independent factor of hyperandrogenic features. Whether or not explainable, functional ovarian hyperandrogenism stands as the primary cause for PCOS. HA brings about premature luteinization, hence causing anovulation and PCO morphology. IR, on the other hand, is associated with systemic metabolic features such as obesity and metabolic disorders [71].

The role of theca cells (TCs) and granulosa cells (GCs) in PCOS could be elaborated from the “two cell, two gonadotropin” theory, which describes the scenario where cholesterol enters the TC through StAR activation. Once LH binds to the TC, the process of steroidogenesis is triggered, eventually producing androgens in the TC as well as estrone and estradiol in the GC. Genetic variants in THADA, INSR, DENND1A, and TOX3 genes, which are linked to PCOS susceptibility and metabolic disorders, have proven to modulate the steroidogenesis in TCs, thus causing HA. Elevated AMH expression owing to excessive follicles induces anovulatory infertility through an effect on reduced sensitivity to follicle-stimulating hormone (FSH) and the inhibition of follicle development. High levels of AMH also affect the SMAD signaling pathway due to increased inhibitory SMADs. The disruption in GC function in turn impacts hormone receptor expression and downstream signaling cascade. Meanwhile, IR is associated with an increased SH2B adaptor protein 3 level, which affects the PI3K-AKT pathway and promotes GC apoptosis and hence ovulation disorders [72].

Since PCOS is a multisystem disorder, there is great diversity in its clinical presentation. To start with body figure, most PCOS women appear to be more obese than the average population, though there is a subgroup with normal BMI called the “lean PCOS”. In terms of the key features of PCOS, including ovulatory dysfunction, hyperandrogenism, and polycystic ovaries, ovulatory dysfunction with oligo-ovulation or anovulation manifests as oligomenorrhea or amenorrhea in 70 to 80% of patients. Oligomenorrhea is defined as a menstrual cycle exceeding 35 or 45 days or less than eight cycles per year. Patients often present to the clinic with complaints of irregular menstruation or infertility. Nevertheless, some women with PCOS experience a normal menstrual cycle. Clinical features of hyperandrogenism include hirsutism, which refers to a male-like hair pattern that could be assessed with Ferriman–Gallwey (mFG) score, with a score over 6 points indicating significant hirsutism [73]. Another common presentation of hyperandrogenism is acne [74]. Finally, the morphology of polycystic ovaries is usually only confirmed by ultrasonography. Other potential clinical manifestations and comorbidities encompass hypertension, diabetes mellitus, dyslipidemia, sleep apnea, depression, etc. [74]. Furthermore, an increased prevalence for nonalcoholic fatty liver has also been proposed [75]. The clinical manifestations are similar in adults and in adolescents with PCOS [76].

## 5. Management of PCOS

There is no curative treatment for PCOS to date. Currently, management strategies include lifestyle and diet modification, medication, and surgical intervention, as listed in Table 2.

### 5.1. Lifestyle and Diet Modification

Lifestyle and diet modification is the first-line treatment for PCOS and has proven to be effective in many studies. In 2019, Siew S Lim conducted a study to review 15 randomized controlled trials (RCTs) to compare women affected by PCOS who underwent lifestyle and diet modification with their counterparts who did not or only underwent minimal modification. Those with a more aggressive intervention were likely to have an improved free androgen index (FAI), a reduced body weight or body mass index (BMI), and an improved secondary reproductive outcome [77]. A paper published in 2021 also suggested that certain dietary interventions—such as keeping a Mediterranean or a ketogenic diet—are beneficial to obesity and IR in women with PCOS [78]. Another study in the same year also recommended a low-calorie and low-GI diet, normalization of sleep, and initiation of daily physical activities for PCOS patients. Moreover, an increased intake of antioxidants like vitamins and minerals could potentially improve chronic physical inflammation and liver steatosis [79]. To define physical activities more precisely, the 2018 PCOS guideline provides a recommendation of at least 150 min of moderate exercise or at least 75 min of intense exercise per week to prevent body weight gain. A minimum of 250 min of moderate exercise or 150 min of strenuous exercise is indicated for further weight loss and regain prevention [80].

### 5.2. Medication of PCOS

Pharmacologic intervention could be applied to various aspects of PCOS, such as improvement in hyperandrogenic manifestations, management of metabolic disorders, ovulation induction, etc. In this section, we aim to provide a review on the drugs of choice for PCOS and the pharmacodynamics that lie behind.

#### 5.2.1. Improvement in Hyperandrogenic Features

Antiandrogens reduce androgen levels and the action of testosterone by binding to androgen receptors, thus ameliorating physical conditions related to androgen excess such as hirsutism. Spironolactone, for example, functions as an androgen antagonist by binding to the androgen receptor. Another frequently applied drug—flutamide—is a nonsteroidal antiandrogen with no progestogenic effects. Finasteride, meanwhile, acts as a 5α-reductase inhibitor that decreases dihydrotestosterone (DHT) production in turn. These are some common options for the treatment of hirsutism [81].

A combination of antiandrogenic drug and another drug such as metformin or an oral contraceptive is often applied for hirsutism. It is important to note that FDA does not approve of antiandrogens use for hirsutism during pregnancy, and that the aforementioned drugs should be strictly avoided in pregnant women [82].

#### 5.2.2. Management of Metabolic Disorders

Metabolic dysregulation is common in PCOS, which is frequently associated with obesity and IR. Once lifestyle and diet modification fails to achieve satisfactory improvements, pharmacological approaches should be considered with frequent selection of metformin and thiazolidinediones (TZDs) like pioglitazone and rosiglitazone [83].

##### Metformin

Metformin is an old, cheap, safe biguanide drug with wide application through its action of improving insulin sensibility. Besides its usage for diabetes mellitus, metformin has been applied in other various fields such as cancers, cardiovascular diseases, and renal diseases. Due to its disease nature of IR, metformin is also a reasonable choice for treating PCOS by way of ameliorating hyperinsulinemia and resultant hyperandrogenism [84].

**Figure 8:**
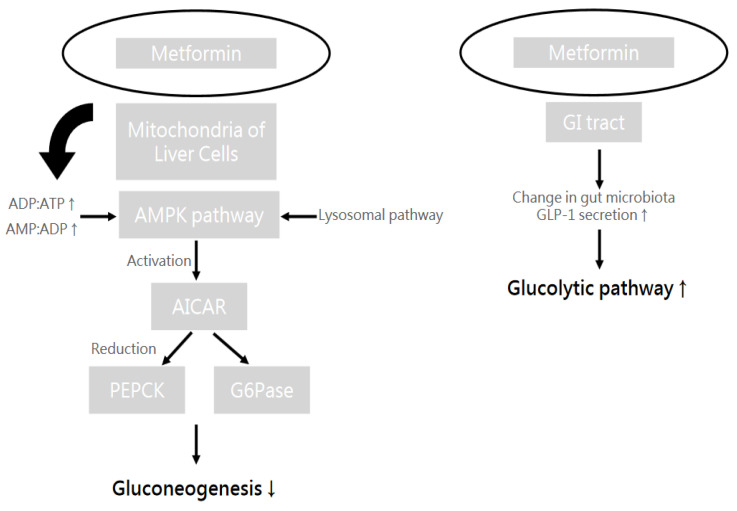
The action mechanism of metformin in the liver and gastrointestinal (GI) tract. Metformin boosts the AMPK pathway in the liver through the lysosomal pathway and an increase (↑) of ADP:ATP and AMP:ADP ratios. A series of reactions can lead to the decrease (↓) of gluconeogenesis. On the other hand, metformin causes a change in gut microbiota in the GI tract, as well as an increase (↑) in GLP-1 secretion, which further enhances (↑) the glycolytic pathway. Finally, the action of metformin results in increased sugar consumption and decreased sugar production.

The complex pharmacological mechanism of metformin has not been fully understood, but there are some possible theories that may provide an explanation. One scenario is that metformin acts on the liver via adenosine monophosphate (AMP)-activated protein kinase (AMPK)-dependent pathways. Metformin accumulates in the mitochondria and inhibits the mitochondrial function. An increase in AMP/adenosine triphosphate (ATP) and adenosine diphosphate (ADP)/ATP ratios activates AMPK. AMPK could be activated through a lysosomal pathway, too. AMPK then activates 5-aminoimidazole-4-carboxamide ribonucleoside (AICAR), which in turn downregulates gluconeogenic enzymes. However, there is still controversy regarding this theory, and there seem to be AMPK-independent pathways involved in the mechanism of metformin. The intestines, for example, may be a target organ for metformin, too. Scholars have proposed several putative mechanisms such as an increased glucagon-like peptide-1 (GLP-1) secretion or a change in the gut microbiota [85]. Figure 8 illustrates the action mechanism of metformin in different sites of human body.

Since PCOS is associated with IR, which is likely to be a contributor to HA, it makes sense that metformin use in PCOS patients provides metabolic benefits. Moreover, metformin may be effective for managing PCOS-related infertility, which will be discussed in the next section [86].

##### Thiazolidinediones (TZDs)

Thiazolidinediones are another group of insulin sensitizers that could treat diabetes mellitus and obesity. More precisely speaking, they are peroxisome proliferator-activated receptor-γ (PPAR-γ) agonists. PPARs are primarily found in tissues characterized by high activity of fat-related metabolism. They regulate downstream gene transcription through ligand-binding. TZDs target PPARs in adipose tissues primarily, which impacts gene expressions that are related to insulin sensitivity, adipokine release, lipid storage capacity of fat tissues, etc. [87,88].

The effects of TZD use in PCOS have been proven for long, referring to pioglitazone and rosiglitazone specifically. They not only improve insulin sensitivity, but also reduce androgen secretion, and may even have a direct ovulatory effect on the ovary though this effect was not well demonstrated in rodent models [89]. Compared to metformin administration alone, dual therapy with metformin plus TZD in PCOS patients seems to have better therapeutic performance in insulin sensitivity and HA, and may encompass an extra benefit in improving lipid metabolism [90].

##### Other Drugs for Metabolic Control

Alternatively, some have suggested the administration of other drugs to manage metabolic disorders in PCOS. In a study published by Antoni J. Duleba, acarbose, naltrexone, orlistat, and statins have been offered as treatment options. Acarbose functions as an α-glucosidase inhibitor in the small intestine that decreases glucose absorption, hence lowering the corresponding serum insulin level. Naltrexone is an opioid antagonist that increases sympathetic activity, which finally leads to a decrease in insulin secretion. Orlistat does not have a significant impact on insulin, but directly reduces fat absorption by acting as a gastric and pancreatic lipase inhibitor. Statins inhibit HMG-CoA reductase and improve lipid profiles. Studies have shown its effects of general metabolic and endocrine improvements in patients with PCOS [91]. Furthermore, other oral antihyperglycemic agents administered in diabetes mellitus may offer metabolic benefits, too. Glucagon-like peptide-1 (GLP-1) receptor analogues, dipeptidyl peptidase-4 (DPP-4) inhibitors, and sodium-glucose co-transporter-2 (SGLT-2) inhibitors are some examples [92].

#### 5.2.3. Fertility Concerns: Ovulation and Contraception

##### Ovulation

One of the leading causes for female infertility though it may be, pregnancy can be achieved when ovulation is resumed. Lifestyle and diet modification is considered to be the initial attempt, followed with pharmacologic intervention, and ultimately assisted reproductive technologies (ARTs) such as in vitro fertilization (IVF) if these frontal measures are still to no avail. It is essential that male factors and tubal infertility have been excluded before medication that targets ovulation induction is administered [93].

Traditionally, clomiphene citrate (CC) is the first-line pharmacologic treatment. The chemical structure of CC resembles a chloroethylene, and only its diphenyl portion is biochemically active. It functions as a competitive antagonist at hypothalamic estrogen receptors. As a result of negative feedback, both the serum level and pituitary sensitivity of GnRH are increased, stimulating FSH and LH secretion and likewise the recruitment and maturation of follicles [94]. Approximately 80% of women with PCOS are sensitive to CC treatment—referring to ovulation rates only, as ovulation is not equivalent to conception—after three cycles of CC administration. For those with CC resistance, a higher dosage may be attempted. An addition of metformin or transition to another drug such as letrozole should also be offered as therapeutic options [95].

Letrozole is a nonsteroidal aromatase inhibitor that has an important role in ER (estrogen receptor)-positive metastatic breast cancer owing to its estrogen-suppressing effect. In women at reproductive age, letrozole can replace CC in infertility treatment. In contrast to CC, it does not disturb the building of the endometrium and cervical mucus. Meanwhile, cases of letrozole-related hepatitis and autoimmune conditions have been reported in women with breast cancer [96,97]. Studies in the recent years have shown that letrozole outperformed CC in PCOS patients regarding ovulation resumption, pregnancy rates, and live birth rates. There is increasing evidence suggesting letrozole instead of CC as the first-line pharmacological treatment for women with PCOS suffering from infertility [98,99].

Aside from improving IR, metformin plays a part in ovulation induction as well. The mechanism is not fully understood but may be related to the reduction in insulin levels to improve hyperinsulinemia [100]. RCTs have suggested that metformin could be used alone for the purpose of correcting anovulation, although such clinical application may be rare. Nevertheless, dual therapy with CC plus metformin has been approved as an effective treatment option [86,101].

##### Contraception

Methods for birth control or menstrual regulation in PCOS patients are similar to those for the general female population, including hormonal and barrier contraception. Hormonal contraception usually refers to the use of oral contraceptives, which not only prevents pregnancy but also helps alleviate HA-related symptoms.

Combined oral contraceptives (COCs) are a common treatment of choice. COCs are composed of an estrogen component and a progesterone component. The progesterone content suppresses GnRH secretion via negative feedback, resulting in a decrease in LH, thus inhibiting ovulation. Estrogen, on the other hand, suppresses FSH and thereby inhibits the growth of a dominant follicle. COCs prevent pregnancies in a way similar to progestin-based contraceptives, but they have a greater benefit for resumption of regular menstrual bleeding [102]. As a part of COC, estrogen also increases sex hormone-binding globulin (SHBG) production, which explains its effect of improving hyperandrogenic features. However, extra caution should be raised regarding adverse events related to hypercoagulability such as venous thrombotic events (VTE). Those who are contraindicated for COC use should consider progestin-based contraceptives or barrier contraception [103,104].

As one COC containing cyproterone acetate and ethinylestradiol, Diane^®^ is commonly used to treat PCOS and hyperandrogenism. Cyproterone acetate is a synthetic steroid with anti-androgenic properties that inhibits the effects of androgens in the body. This helps reduce symptoms of hyperandrogenism such as hirsutism (excessive hair growth), acne, and seborrhea. As described above, ethinylestradiol, a synthetic estrogen, complements cyproterone acetate by increasing SHBG levels in the blood. Higher SHBG levels decrease the amount of free androgens, thereby reducing their activity. Ethinylestradiol also provides contraceptive effects and stabilizes the menstrual cycle, which is often irregular in PCOS patients. The combination of these two hormones in Diane^®^ helps regulate hormonal imbalances typical in PCOS, reducing ovarian androgen production and preventing ovulation. This dual action alleviates symptoms associated with hyperandrogenism and PCOS, improving the overall quality of life for affected women. By addressing both the androgen excess and menstrual irregularities, Diane^®^ is an effective treatment option for managing PCOS and its related manifestations.

### 5.3. Surgical Intervention

Surgical management remains to be a treatment option for PCOS, albeit not very popular. The most common procedures to enhance ovulation and pregnancy rates are laparoscopic ovarian drilling (LOD) and transvaginal hydrolaparoscopy (THL). During LOD, surgeons create openings on the ovarian capsules with laser energy or electrodes. The concept of THL is similar but via a different route. It starts with entering the peritoneal cavity through the cul-de-sac using a Veress needle. The peritoneal cavity is then instilled with normal saline. Finally, a channel is inserted into the cul-de-sac, allowing for the scope to enter the peritoneal cavity and complete ovarian drilling. The treatment effects (pregnancy and delivery rates) seem to be similar between LOD and THL. Some have stated that THL is associated with less adhesion formation when compared with LOD [105,106].

To date, the mechanism of ovarian drilling is yet to be unveiled. One possible theory is that the damaged ovarian stroma produces less androgen, which results in a significantly lower level of total and free testosterone. It has also been found that ovarian drilling leads to an eventually reduced LH and the pituitary responsiveness to GnRH [105,107]. For those who are resistant to pharmacological treatment and seek for conception, surgery could be offered as an option after explaining the risks of anesthesia and surgery to the patient.

In addition to fertility concerns, surgical intervention may be considered for body weight control for PCOS patients with morbid obesity and unsatisfactory treatment effects from traditional pharmacotherapy. While it is still experimental, some studies have shown the benefits of bariatric surgery for women with PCOS who suffer from obesity and subfertility. Bariatric surgery serves as an effective means to reduce body weight, resume menstrual regularity, and diminish hyperandrogenic manifestations [108,109].

In addition, women suffering from hirsutism and especially facial hair may seek hair removal. A relatively conservative way is laser treatment, which brings somewhat satisfactory results even though the effects seem to be poorer than in those without PCOS [110]. A surgical approach could be carried out via electrolysis, where thermolysis is performed by inserting a needle into and destroying the hair follicle with a direct current. Shaving is recommended before surgery [111].

## 6. Conclusions and Future Perspectives

PCOS is a rather prevalent condition affecting reproductive-aged women globally with lifelong impacts, manifesting as HA, OD, and PCO morphology, and it is most frequently defined by the Rotterdam criteria. Owing to the complexity and diversity of the disorder, its long-term risks, pathophysiology, and ideal management approaches remain a big challenge. Various factors may contribute to the mechanism of PCOS, including both “nature” and “nurture”. A deeper understanding of the etiology and pathophysiology of PCOS long has been a main focus for research, since it may shed light on the design of an optimal management strategy. Until now, current management often puts an emphasis on short-term concerns and symptom relief due to the lack of specialized pharmaceutical regimens.

Hence, it seems plausible that a better grasp of the delicate causative relations between HA, IR, and PCOS may provide a finer picture of targeted drug development. In our article, we aimed to review the details of potential cellular and molecular aspects regarding the mechanism and management PCOS. While the complexity of PCOS has partially been unveiled, an optimal treatment is yet to be identified. Being aware of the complications associated with PCOS—such as metabolic disorders, increased risk of future endometrial hyperplasia and malignancy [112], and features related to HA—nevertheless necessitates early detection and screening, regular follow-up, and suitable treatment for women with PCOS to improve the prognosis and outcome.

Future research may focus on the prevention of PCOS, and also potential treatment strategies that have not been well established yet, such as the augmentation of intestinal microbiome regarding gut dysbiosis related to PCOS [8]. If more genetic and molecular details of PCOS are disclosed, designs for new treatment strategies may emerge as well. This review aimed to provide an in-depth examination of the etiology, pathophysiology, diagnosis, and management of PCOS, with a focus on its molecular and cellular aspects. Nevertheless, many unknown aspects of PCOS, including detailed mechanisms of action, along with the safety and effectiveness of the treatment, warrant further investigation.

## Figures and Tables

**Table 1:** A comparison of different diagnostic criteria for PCOS.

Feature	The NIH Criteria	The Rotterdam Criteria	The AE-PCOS Criteria
Hyperandrogenism (HA)	Biochemical or clinical evidence	Biochemical or clinical evidence	Biochemical or clinical evidence
Ovulatory dysfunction (OD)	Chronic oligo-anovulation	Chronic oligo-anovulation	Chronic oligo-anovulation
Polycystic ovarian morphology (PCO)		At least 12 follicles at the size of 2–9 mm in diameter or an ovarian volume > 10 cm^3^ in one or both ovaries	Polycystic ovarian appearance on imaging
Features required for diagnosis	Both HA + OD	2 of 3	HA + at least one other criteria
Phenotypes	HA + OD + PCO	HA + OD HA + PCO OD + PCO HA + OD + PCO	HA + OD HA + PCO HA + OD + PCO

**Table 2:** The management of PCOS.

Measures	Details
Lifestyle and diet modification (first-line treatment)	Engagement of physical activities Low-calorie, low-GI, Mediterranean, and ketogenic diet Vitamin and mineral intake
Medication	Antiandrogens, e.g., spironolactone, flutamide, and finasteride Medication for metabolic disorders, e.g., metformin, thiazolidinediones, acarbose, naltrexone, orlistat, and statins Induction of ovulation: e.g., clomiphene citrate, Letrozole, and metformin Contraception: e.g., combined oral contraceptives with or without cyproterone
Surgical intervention	Enhancement of ovulation: e.g., laparoscopic ovarian drilling, transvaginal hydrolaparoscopy Bariatric surgery Hair removal
